# Sexual mixing and the risk environment of sexually active transgender women: data from a respondent-driven sampling study of HIV risk among transwomen in San Francisco, 2010

**DOI:** 10.1186/1471-2334-14-430

**Published:** 2014-08-06

**Authors:** Erin C Wilson, Glen-Milo Santos, H Fisher Raymond

**Affiliations:** San Francisco Department of Public Health, San Francisco, California USA

## Abstract

**Background:**

Research on the sexual networks of transwomen is central to explaining higher HIV risk for this population. This study examined HIV risk behaviors and sexual mixing patterns of transwomen by demographic and HIV-related risk behaviors.

**Methods:**

Data were obtained from a 2010 study of HIV risk for transwomen in San Francisco. Assortativity by race, partner type, HIV serostatus, and IDU across sexual networks was calculated using Newman’s assortativity coefficient (NC). Multivariable generalized estimating equations (GEE) logistic regression models were used to evaluate associations between unprotected anal intercourse with race and HIV serostatus, partner-IDU status and relationship type discordance while adjusting for the HIV status of transwomen.

**Results:**

There were 235 sexually active transwomen in this study, of whom 104 (44.3%) were HIV-positive and 73 (31.1%) had a history of injection drug use. Within the 575 partnerships, African American/black and Latina transwomen were the most racially assortative (NC 0.40, 95% CI 0.34-0.45, and NC 0.43, 95% CI 0.38-0.49, respectively). In partnerships where the partner’s HIV status was known (n = 309, 53.7%), most transwomen were in sexual partnerships with people of their same known serostatus (71.8%, n = 222). In multivariable analyses, unprotected anal intercourse was significantly associated with primary partners, having a sexual partner who was an injection drug user, and sexual partner seroconcordance.

**Conclusions:**

Public health efforts to reduce transwomen’s HIV risk would likely benefit from prioritizing prevention efforts to risk reduction within IDU-discordant and primary partnerships, determining risks attributable to sexual network characteristics, and actively addressing injection drug use among transwomen.

## Background

A systematic review of HIV research in the US found that transgender women (transwomen) engage in high rates of unprotected receptive anal intercourse, which may explain elevated rates of HIV [1]. Findings from epidemiological research on HIV suggests that members engaging in serodiscordant partnering, or sex between partners with different HIV statuses, is key to maintaining and transmitting HIV within populations at risk and their sexual partners [2]. Past studies of transwomen have found that sexual risk behavior is highest in primary sexual partnerships [3–5]. However, one recent study found that risk for HIV was highest for transwomen who did sex work, possibly due to the higher volume of sexual partnerships by choice or by assault that increased chances for exposure to HIV [6]. Transwomen have also been found to be more likely than other populations at risk for HIV to have serodiscordant partnerships, though partner serodiscordance did not significantly influence engagement in unprotected receptive anal intercourse [7]. HIV risk may, in part, be explained by the behaviors specific to particular partner types and the serostatus of those partners.

Transwomen may also be at risk for HIV due to substance use. A number of studies have demonstrated elevated use of illicit drugs among transwomen [1, 8]. A recent population-based study of transwomen found that testing positive for HIV was significantly related to methamphetamine use, methamphetamine use before or during anal intercourse, and at least weekly methamphetamine use [9]. Concerns about HIV transmission via injection drug use among transwomen have been related to needle sharing practices, injection of hormones or other fillers (i.e. illicit “silicone”) and injection drug use behaviors of sexual partners [10–12]. Risk related to injection hormones is mixed as one study found that a combination measure of injection drug and hormone use to be related to self-reported HIV infection, while another recent study of injection of fillers (i.e. illicit substances used to feminize transwomen’s appearance) found no association with an HIV positive test [8, 13]. A population-based study of transwomen in San Francisco found that 32.5% had ever injected substances not prescribed by medical professionals and 11.8% had injected in the past 12 months [14]. In the same study, injection drug use in the past 12 months was one of only three factors that remained significantly associated with HIV infection. Transwomen who inject drugs may be at high risk for HIV from non-sterile injection equipment, and from sexual transmission, as this mode of transmission accounts for a substantial number of incident infections in some studies [15, 16]. However, data are missing on HIV risk related to sex with injection drug users among transwomen, thus it is difficult to discern whether IDU-related risk among transwomen is due to injection drug use practices of transwomen, their sexual partners who are IDU, or both.

By comparison, race has been an important sexual mixing factor as it relates to HIV risk among MSM [17]. Bohl et. al’s [17] article on racial disparities in HIV found that the sexual networks of African American/black MSM account for their higher prevalence of HIV compared with non-African American/black MSM. This study provided compelling evidence that sexual networks rather than individual characteristics or behaviors explained significantly higher risk of HIV for African American/black MSM [17]. The systematic review of HIV among U.S. transwomen found that the weighted mean HIV prevalence for African American/blacks was 56.3%, which was almost twice as high as that found among White or Hispanic transwomen [1]. An important next step in the investigation of HIV risk for transwomen is to better understand risk via their sexual networks. To fill a void in what we know about the sexual partnerships of transwomen, we conducted an analysis of demographic and risk factors of the sexual networks of sexually active transwomen in San Francisco. We also evaluated which sexual mixing factors by race, partner type, IDU status, and HIV status were correlated with HIV-related sexual risk behaviors.

## Methods

### Respondent-level variables - transwomen participants

Data for this analysis are taken from a study of transgender women that took place in 2010 (TEACH- the Transgender Empowerment and Community Health study). Transgender women were recruited using respondent driven sampling (RDS) in which 11 initial seed participants recruited a total final sample of 314 transwomen [18]. Individuals were eligible for the study if they (1) self-identified as transwomen, (2) were age 18 years or older and (3) reported living in San Francisco. Each study participant was screened for study eligibility prior to enrollment. Eleven seeds diverse in respect to race/ethnicity, income, education, and age were selected as the initial recruits. Recruits could be anyone in their social network who seeds (or recruiters) thought was eligible for the study. All participants were remunerated $10 for recruiting peers who were eligible and enrolled into the study (see Rapues et al., 2013 for more information on seeds). All data on HIV status were collected by either self-report and/or through an HIV assay. Respondents who were HIV-positive were able to refuse the HIV test with no repercussions for receiving an incentive.

Participants were asked to report on up to 5 sexual partners from the past six months, including each partner’s age, gender identity, race/ethnicity, what type of partner each person was (i.e. primary, casual or commercial), where the participant met each partner, sexual behavior, HIV status and whether that person was an injection drug user. We created a dataset of all the sexual partnerships of TEACH participants for the past six months. Those that did not report having a sexual partner in the last six months, those with missing data on HIV-serostatus, and those who were HIV-positive but with an unknown positive status at the time of participation were excluded from this analysis.

### Statistical analysis

We analyzed sexual partner mixing by race, HIV serostatus, and explored partner-level characteristics including relationship type and IDU status. Consistent with surveillance and other sexual mixing studies, partner data analyzed for this study are egocentric and therefore based solely on data reported by transwomen participants, not from their partners [19]. Sexual risk in TEACH was assessed by asking a number of questions about characteristics and behaviors of the last 5 sexual partners of transwomen and by asking transwomen what sexual behaviors they engaged in with each of these partners. This strategy is employed nationally in the U.S. and in global HIV surveillance efforts to enhance recall and obtain specificity in recent sexual behaviors of populations most at risk for HIV [20].

Transwomen were also asked to identify the race, HIV status, and injection drug use status of their last 5 sexual partners. Partner types were categorized as main, casual and commercial. Main partners were defined as, “someone who is your primary sexual partner and you feel committed to (boyfriend, lover, husband, girlfriend, wife)”. Casual partners were defined as, “someone you have sex with, but don’t feel committed to or don’t know very well”. Commercial partners were defined as, “someone you had sex with in exchange for things like money or goods”.

Sexual mixing in HIV status or serodiscordance was defined as a difference in reported or tested HIV status of the transwomen participant and the reported HIV status of the sexual partner, as has been done in studies of MSM [21, 22]. We excluded 47 partnerships where the HIV status of the transwoman participant was unknown. In the case that a transwoman participant was HIV positive and reported her partner’s HIV status as negative, and vice versa for a transwoman who was HIV-negative and reported her partner as being HIV-positive, the partnership was categorized as serodiscordant. Transwomen’s partnerships with those of an unknown HIV status were also categorized as serodiscordant, consistent with prior literature [23, 24]. We calculated assortativity by race, partner status (main, casual, or commerical), HIV serostatus, and IDU between transwomen and their sexual networks using Newman’s assortativity coefficient. Newman’s assortativity coefficients ranges from −1 to 1; negative coefficients correspond with disassortativity or a higher-degree of mixing, while positive coefficients correspond with assortativity or a lower-degree of mixing [25]. A study of MSM in San Francisco over three waves of data collection from 2004–2011 found slightly assorative mixing by race overall at r = 0.08 in 2004, but highly assortative partnering for African Americans/blacks (r = 0.44) [26]. Based on this study and the work of Doherty et al. [27], we have categorized mixing coefficient values ≥ 0.35 as assortative, 0.15–0.34 as moderately assortative, and < 0.15 as disassortative.

### Multivariable analysis of engagement in sexual risk behavior

In the primary outcome multivariable analysis, we evaluated the associations between UAI with sexual partners and discordance in race and HIV serostatus, as well as partner-IDU status and relationship type, while adjusting for the HIV status of transwomen. We used multivariable generalized estimating equations (GEE) logistic regression models that accounted for correlation between the multiple partnerships of each study participant to estimate odds ratios and 95% confidence intervals.

Analyses were conducted using STATA 12.0. Data adjustments to make inference to the population from which the sample was drawn were not made because the sample was stratified for this analysis and the exclusion of 79 participants eliminates the ability to assure equilibrium in the sample. Therefore, findings from this study describe findings from this dataset alone and may not be generalizable to the San Francisco transwoman population overall.

This study received human participants review and approval from the University of California, San Francisco’s Committee on Human Research.

## Results

### Participants demographics

Of the 314 TEACH participants, 15 refused to take an HIV test and 64 did not have any sexual partners in the past six months, thus leaving 235 transwomen for our analysis. Among these sexually active transwomen 44.3% (n = 104) were HIV-positive (Table 1). Of the 235 sexually active transwomen, 72.3% (n = 170) reported having five or fewer sexual partners. The range of sexual partnerships per transwomen was 1–500, with a median number of three (inter-quartile range [IQR]: 1–6) sexual partners and a mean of 11 (standard deviation: 39.01). However, not all sexual partnerships were included in this analysis as participants were only asked about characteristics of up to five individual sexual partners. Within this dataset, a respondent may have had her data repeated, but each partnership was unique.

**Table 1:** **Characteristics of sexually active transwomen participants (n = 235) and their sexual partners in the last 6 months (n = 575)**

Transwomen participant demographics and behaviors	n ***mean***	(%) ***(SD)***
Age, *mean (SD)*	*41*	*(10.7)*
Gender Identity		
Female	112	(47.7)
Transfemale	123	(52.3)
Race/Ethnicity		
African American/black	73	(31.1)
Asian or Pacific Islander	15	(6.4)
Latina/Hispanic	72	(30.6)
White	36	(15.3)
Other	39	(16.6)
Injection drug use		
No	162	(68.9)
Yes	73	(31.1)
HIV Status		
Negative	131	(55.7)
Positive	104	(44.3)
Any Unprotected Anal Intercourse with sexual partners		
No	147	(62.6)
Yes	88	(37.5)
**Sexual partner demographics and behavior**		
Age, mean (SD)	34.8	(10.6)
Gender Identity		
Male	537	(93.4)
Female	17	(3.0)
Transwoman	13	(2.3)
Transman	3	(0.5)
Other/Did not specify	5	(0.9)
Race/Ethnicity		
African American/black	183	(31.8)
Asian or Pacific Islander	15	(2.6)
Latino/Hispanic	134	(23.3)
White	195	(33.9)
Other	48	(8.4)
Relationship with participant		
Primary	151	(26.3)
Casual	322	(56.0)
Commercial	102	(17.7)
Meeting site		
On the street, park or public place	225	(39.1)
Introduced by friends	67	(11.7)
Internet	62	(10.8)
Bar	57	(9.9)
Other	164	(28.5)
Injection drug use		
No	520	(90.4)
Yes	55	(9.6)
HIV Status		
Positive	241	(41.9)
Negative	68	(11.8)
Unknown	266	(46.3)
Same HIV-status as participant		
No	353	(61.4)
Yes	222	(38.6)
Same race as participant		
No	365	(36.5)
Yes	210	(63.5)
Unprotected Anal Intercourse with participant		
No	452	(78.6)
Yes	123	(21.4)

Over 60% of the sample was transwomen who were either Latina (30.6%) or African American/black (31.1%). Whites made up 15.3% of the sample, 16.6% were “Other” and only 6.4% of our sample was Asian or Pacific Islander (API). Transwomen split almost evenly into identifying as transgender or female (52.3% and 47.7%, respectively). The median age of transwomen in this analysis was 42 (IQR 32–48) and the mean was 41 (SD 10.7). Most transwomen did not have a lifetime history of IDU (n = 162, 68.9%), but a sizeable proportion did report being IDU (n = 73, 31.1%).

### Partners demographics

There were a total of 575 partnerships analyzed from the 235 sexually active transwomen respondents. The characteristics of these partners are summarized in Table 1. Most partners of transwomen were between the ages of 26–43 (50%). The mean age of partners was 34.8 (SD 15). The majority of partnerships were with men (n = 537, 93.4%), and the most common place where transwomen met their sexual partners was on the street (n = 225, 39.1%). The majority of sexual partnerships were casual (n = 322, 56%), while 26.3% (n = 151) were with primary partnerships and 17.7% (n = 102) were commercial relationships.

### Sexual partners and mixing

#### HIV

Among transwomen with a known HIV status, almost half of transwomen’s sexual partnerships were with partners of an unknown HIV status (46.3%, n = 266). Of those who knew their partner’s HIV status (n = 309, 53.7%), transwomen were in more sexual partnerships with people of their same known serostatus (36.5%, n = 222). Among sexual partners of transwomen with a known HIV serostatus, the Newman’s coefficient was 0.40 (0.33-0.47), suggesting that transwomen were assortative in their sexual partnering with people of the same serostatus. Excluding partnerships where the HIV status was unknown for partners (n = 266), 25% of partnerships were between HIV-positive transwomen and HIV-negative partners (n = 78, 24.3%). Only 3% of partnerships were between HIV-negative transwomen and an HIV-positive sexual partner (n = 9).

#### IDU

Most transwomen did not ever inject drugs, though a sizeable portion did inject drugs (n = 73, 31.1%). Similarly, most transwomen reported that their partners did not inject drugs (n = 520, 90.4%), while 9.6% of partners (n = 55) reportedly did inject drugs. The Newman’s assortativity coefficient for IDU was 0.23 (95% CI 0.19- 0.26), suggesting that transwomen were moderately more likely to partner with people of the same IDU status. Sexual partnerships where non-IDU transwomen had partners who were IDU made up 13% of IDU-discordant partnerships (n = 19), while sexual partnerships where IDU transwomen had non-IDU partners made up the other 87% (n = 123). Therefore, more transwomen were IDUs in sexual partnerships where IDU status was mixed.

#### Race

The majority of sexual partners of transwomen were White (195 of 575 partners; 33.9%), African American/black (183 of 575 partners; 31.8%), or Latino (134 of 575; 23.3%) (Table 1). However, when assessing partnerships on a race-by-race basis, African-American/black, Latino, and White transwomen most commonly reported having sexual partners of the same race. Sexual partners of White transwomen were 47% White (n = 43), followed by African-American/black 26% (n = 24), and Latino 13% (n = 12). Sexual partners of African-American/black transwomen were 60% African-American/blacks (n = 106), followed by White 29% (n = 40) and Latino 8% (N = 15). Sexual partners of Latino transwomen were 49% Latino (n = 100), followed by white 26% (n = 53) and African-American/black 11% (n = 24). The overall Newman’s assortativity coefficient for race was .26 (95% CI 0.22-0.30), suggesting that transwomen were moderately assortative by race. When distilled by race, the assortativity coefficient by Asian race was 0.03 (95% CI 0.01-0.05), by African American/black was 0.40 (95% CI 0.34-0.45), by Latino was 0.43 (95% CI 0.38-0.49) and by White was 0.11 (95% CI 0.06-0.15). Thus, African American/black and Latina transwomen were more assortative than Whites and Asians, and had sexual partners of the same race more often that transwomen of other races.

#### Prevalence and multivariate analysis of engagement in sexual risk behavior

Among transwomen, 88 (37.5%) reported having UAI with at least one sexual partner. Transwomen reported having UAI in 123 sexual partnerships (21.4% of 575 partners). The results of the GEE multivariable logistic regression model examining the association of sexual mixing factors with unprotected anal sex are summarized in Table 2. Results indicate that compared to primary partners, transwomen had an adjusted odds of UAI that was 0.23 (95% CI 0.15-0.36, p < 0.001) and 0.20 (95% CI 0.10-0.43, p < 0.001) lower with casual and commercial partners, respectively. Moreover, transwomen had an adjusted odds of UAI of 0.55 (95% CI 0.35-0.87, p < 0.001) lower with HIV serodiscordant partners compared to seroconcordant partners. In addition, transwomen had an adjusted odds of UAI that was 2.66 (95% CI 1.43-4.97, p < 0.001) greater with sexual partners who were IDU than with non-IDU. The odds of engaging in UAI were not significantly different between same race and different race partners of transwomen (aOR 0.77, 95% CI 0.50-1.21, p = .40).

**Table 2:** **Prevalence and Multivariable GEE logistic regression model on Partner-level correlates of Unprotected Anal Intercourse**

		UAI with partners				Crude			Adjusted*		
		No	(%)	Yes	(%)	OR	95% CI	P-value	AOR	95% CI	P-value
Partner Characteristics	Same race partners	252	(74.1)	88	(25.9)	1	-	-	1	-	
	Different race partners	200	(85.1)	35	(14.9)	0.80	0.54-1.20	0.28	0.77	0.50-1.21	0.26
	Primary partner	87	(57.6)	64	(42.4)	1	-	-	1	-	-
	Casual partner	279	(86.6)	43	(13.4)	0.21	0.13-0.32	<0.001	0.23	0.15-0.36	<0.001
	Commercial partner	86	(84.3)	16	(15.7)	0.18	0.09-0.37	<0.001	0.20	0.10-0.43	<0.001
	Non-IDU partners	420	(80.8)	100	(19.2)	1	-	-	1	-	-
	IDU partners	32	(58.2)	23	(41.8)	2.44	1.37-4.33	0.002	2.66	1.43-4.97	0.002
	HIV seroconcordant partners	154	(69.4)	68	(30.6)	1	-	-	1	-	
	HIV serodiscordant partners	298	(84.4)	55	(15.6)	0.43	0.29-0.64	<0.001	0.55	0.35-0.87	0.01

Notes: *Model adjusted for participant-level characteristics in table above and individual-level co-variables (e.g. Age and HIV-status of TEACH participant). GEE models adjusted for clustering of partner-level data by TEACH participant.

## Discussion

The goal of this analysis was to determine if there were significant sexual mixing patterns in addition to individual risk behaviors to help understand high HIV risk among transwomen. In the final analysis we found that UAI was significantly associated with having a sexual partner who was an injection drug user, primary partner, and/or HIV seroconcordant. Transmission of HIV from injection drug users to sexual partners who are uninfected may be a source of HIV risk for transwomen. Sexual transmission of HIV from seropositive IDUs to non-injecting sexual partners is extremely likely, and more risky than that associated with sharing needles [25]. In this study, only 10% of partners were IDU, but that represented 55 sexual encounters for transwomen, thus mixing with individuals who are injection drug users remains an important area of investigation for understanding transwomen’s risk for HIV.

These data also support prior studies that have shown higher rates of UAI within primary partnerships [3, 4]. Of all three partner types - primary, casual and commercial - primary partners have consistently been the type in which transwomen engage in the highest risk behavior, which is consistent with the literature for MSM, IDU and heterosexual men and women [3, 4, 28–32]. Yet transwomen in this analysis were also less likely to engage in UAI with serodiscordant partners. Many of the partnerships analyzed as serodiscordant were those in which transwomen did not know the HIV status of their sexual partner, which made up almost of half of sexual partnerships. The finding on partnerships of unknown HIV status is higher than recent research with Peruvian transwomen and MSM showing that one quarter of partners were people of unknown serostatus [33]. Yet findings that transwomen were significantly less likely to engage in UAI with partners of a different serostatus is consistent with other research findings [7], and may also be a sign that transwomen are serosorting. Available data on the effects of serosorting on HIV transmission are equivocal; some studies suggest protective benefits [34], while some have found increased risk for transmission due to serosorting [12]. Given the disproportionate impact of HIV among transwomen, it would be prudent to inform transwomen about possible limitations of serosorting in reducing HIV risk. Also different, most serodiscordant partnerships in our study were characterized by transwomen living with HIV. As has been found in research with HIV-positive MSM, transwomen already living with HIV many may be choosing HIV-negative partners to protect from super-infection and other sexually transmitted diseases [35]. Alternatively, this finding may represent the overall high prevalence of HIV among transwomen and the high likelihood of having a HIV-negative partner.

One risk we did not observe in this study was an association between same race sexual partnerships and risk for HIV via elevated rates of engagement in UAI. Highly interconnected networks among African American/black MSM have been a driver of HIV in the population [1, 14, 36]. African American/black transwomen have almost twice the rate of HIV as the overall transwomen population [18]. Assortativity was significantly higher for African American/black and Latina participants and engagement in UAI was not significantly different. Thus, findings of assortativity on race alone may mean highly interconnected sexual networks with those of their same racial/ethnic identities are important for understanding higher HIV risk among African American/black and Latina transwomen. Given the lack of significant differences in engagement in sexual risk behavior, this study provides evidence that same-race partnerships may be a driver of HIV risk for transwomen. More work needs to be done to see if transwomen are facing the same risks for HIV as African American/black MSM that was attributed to sexual networks despite low engagement in sexual risk behaviors.

Finally, data from this analysis point to a diversity of transwomen’s choices in sexual partners by race. Though the largest proportion of sexual partners were the same race as the transwomen participants, up to 70% of partners were of a different race. The only known study of the main male partners of transwomen in which a convenience sample was used to recruit participants found mostly African American/black male partners [37]. The difference in findings on the race of sexual partners between studies may be due to the population-based sampling conducted for this study that points to diverse rather than limited sexual mixing patterns among transwomen overall.

There are a number of limitations to this study. Data were gathered based on self-report, which may be inherently biased due to social desirability. Demographic and behavioral factors of sexual partners were also reported by transwomen respondents and not the partners themselves. Therefore, the characteristics and behaviors of sexual partners may be inaccurate due to biases of the participant, inaccurate knowledge and recall bias. Though these data are taken from a study employing respondent driven sampling, the exclusion of participants who did not recently have a sexual partner may make these data less generalizable to the overall transwoman population in San Francisco.

## Conclusions

These data point to important next steps in research and prevention. Past prevention research has mostly targeted transwomen, and these data suggest that serving one side of the risk equation may not be enough to curb the epidemic within this hard hit population [38]. Indeed, it may be the lack of prevention resources provided to the sexual partners of transwomen that has contributed to the persistent high risk for HIV among transwomen, thus prevention messaging must be designed and targeted to sexual partners too. Research to better understand the considerations of transwomen when selecting sexual partners, such as the convergence with IDU sexual networks, may contribute to more targeted HIV and IDU prevention and harm reduction strategies that impact transwomen’s own drug use behavior and selection of partners who are IDUs [11]. Interventions that work with transwomen to identify ways to ask about the serostatus of their sexual partners are also warranted. Serosorting is only protective when two individuals can make safe sex decisions based on known HIV status. The promise of home testing and instant HIV testing may help further such efforts and should be considered for distribution among transwomen and for their sexual partners. Finally, future longitudinal research with transwomen is also needed so analyses can better determine risks among incident cases instead of extrapolating findings from cross sectional data within high prevalence samples.
